# Effect of Thermal Aging on the Microstructure and Mechanical Properties of ER308L/Z2CND18.12N2 Dissimilar Welds

**DOI:** 10.3390/ma16227119

**Published:** 2023-11-10

**Authors:** Hongmin Ju, Jing Liu, Shiwei Zhuo, Yanli Wang, Shilei Li

**Affiliations:** State Key Laboratory for Advanced Metals and Materials, University of Science and Technology Beijing, Beijing 100083, Chinag20199190@xs.ustb.edu.cn (J.L.); s20161255@xs.ustb.edu.cn (S.Z.)

**Keywords:** reactor structural materials, dissimilar metal weld, microstructure, mechanical properties, nanoindentation, thermal aging

## Abstract

A multi-analytical approach was used to investigate the effect of thermal aging on the microstructure and mechanical properties of ER308L/Z2CND18.12N2. The results demonstrated that fractures occurred preferentially on the ER308L side. Z2CND18.12N2 exhibited superior fracture toughness compared to ER308L regardless of thermal aging time. The ultimate tensile strength significantly increased from 564.5 MPa in the unaged condition to 592.7 MPa to MPa after thermal aging and the fracture mode changed from ductile fracture into a ductile + quasi-cleavage fracture. The fusion zone (FZ) with the chemical composition gradient was about 40 μm from the Z2CND18.12N2 to ER308L. After thermal aging, spinodal decomposition and G-phase precipitation were observed for the first time in the ferrite phase of the FZ. Moreover, the hardness presented the following trend: FZ > ER308L > Z2CND18.12N2. The hardness of the ferrite phase dramatically increased from 6.13 GPa in an unaged condition to 8.46 GPa in a 10,000 h aged condition.

## 1. Introduction

Duplex stainless steels with austenite and ferrite microstructures are materials of great importance in pressurized water reactors (PWRs) due to their excellent corrosion resistance and good weldability [1]. The ferrite phase improves the strength as the hard phase of duplex stainless steels; nevertheless, the ferrite phase is susceptible to thermal aging effect during long-term service at around 300 °C [2]. Thermal aging induces spinodal decomposition into α′ (Cr-rich) and α (Fe-rich) phases in the ferrite phases of duplex stainless steels at temperatures ranging from 280 to 500 °C as the primary mechanism. Moreover, the possible precipitation, G-phase enriched in Si, Ni, and Mn, may be observed during the thermal aging process, but this depends on the content of Si, Mn, Mo, Cr, etc., in duplex stainless steels [3,4,5]. In addition, carbide or solute clusters may also be observed in ferrite or the phase boundaries after thermal aging [1,6,7]. After thermal, these microstructural evolutions respond to the degradation of mechanical properties, especially the loss of fracture toughness and impact toughness, i.e., thermal aging embrittlement [2,8]. Furthermore, diffusion-assisted chemical redistribution results in the chemical variation of dissimilar metal-weld interfaces decreasing the corrosion resistance, which highlights the structural integrity problems of relevant components in PWRs [9,10].

Dissimilar metals welded joints (DMWJs) of ER308L/Z2CND18.12 N2 are the most important structural components in the primary coolant pipe safe-ends of the CPR1000 reactor [1,11]. DMWJs comprise heterogeneous materials with inhomogeneous geometry and metallurgy. In addition to the synergistic effects of high temperature, high pressure, fluid erosion, and corrosion, neutron irradiation degrades the performance continuously [11,12,13,14,15,16]. During the welding process of dissimilar metals, there is usually an element dilution zone between the base metal and the weld metal, such as Mo, Cr, Si, etc., which also affects the service performance of the whole DMWJs [17,18]. Radiation-induced segregation (RIS) depletion in Cr is accordance with Mo depletion (RIS) [19]. The content of Mo significantly influences the content, distribution, and morphology of ferrite, which affects the impact energy and the proportion of cleavage features [4]. The increase in Ni content will accelerate the aging of ferrite in duplex stainless steels, which results in the decrease in mechanical properties and corrosion resistance [5]. Therefore, it is critical to comprehensively explore the mechanical properties of DMWJs after thermal aging. In recent years, various researchers have focused on the relationship between the changes in mechanical properties and the thermal aging of individual DMWJs materials [20,21,22]. However, few studies are concerned with the overall service performance of DMWJs. As the fusion zone (FZ) between the two materials of DMWJs, the ferrite in the fusion zone has not been studied in regard to whether spinodal decomposition occurs during the thermal aging process or how its microstructure and mechanical properties change. At the same time, determining the weak points of DMWJs is essential for practical research.

In the present work, the dissimilar ER308L/Z2CND18.12N2 welds were explored in terms of the relevance between the microstructure evolution and mechanical properties with a nano-indenter after thermal aging at 400 °C for 10,000 h. Microstructure changes in the aged ferrite were observed via transmission electron microscopy and atom probe tomography. As far as we know, this is the first time that the relevance between hardness and microstructure of ferrite after thermal aging in the fusion zone of the dissimilar ER308L/Z2CND18.12N2 welds has been investigated.

## 2. Materials and Experimental Procedures

The ER308L/Z2CND18.12N2 samples used in this study were taken from the primary circuit safe-end welds of CPR1000 PWRs (China National Nuclear Corporation, Beijing, China). The ER308L (Avesta, Stockholm, Sverige) stainless steel layers were deposited on the Z2CND18.12N2 (Dongbei Special Steel Group Co., Ltd., Dalian, China) forged stainless steel via multi-pass submerged arc welding (SAW) without post-weld heat treatment to obtain the DMWJs. The corresponding SAW parameters have been reported previously [23,24]. The chemical compositions of the ER308L and Z2CND18.12N2 were examined using electron probe microanalysis, as shown in Table 1. The aged specimens underwent an isothermal ageing progress at 400 °C for as long as 10,000 h. All samples were arranged through mechanical polishing with approximately a 0.05 μm colloidal silica, followed by electrolytic etching via 0.05 mol oxalic acid solution at 6 V for 65 s. The optical micrographs (OM) were characterized using a confocal laser scanning microscope (CLSM, LEXT OLS4160, Olympus, Tokyo, Japan). Element distribution in the DMWJs was characterized using electron probe microanalysis (EPMA, 8050G, Shimadzu, Tokyo, Japan). All specimens were subjected to X-ray diffraction (XRD, DMAX-RB, Rigaku, Tokyo, Japan) to identify phases. The micromechanical properties across the DMWJs were studied via a Berkovich tip nano-indenter (Nano Indenter DCM, MTS, KLA, Milpitas, CA, USA) with a 500 nm indentation depth. The loading and unloading speeds of nanoindentation tests are 1 nm/s. The microstructure in the fusion boundary zone was observed via field emission transmission electronic microscopy (TEM, G2F30, FEI Tecnai, Hillsboro, OR, USA). The ferrite phase decomposition in the FZ was investigated via atom probe tomography (APT, LEAP 5000XR, Cameca, Paris, France). TEM and APT specimens were extracted from the FB region with a thickness of approximately 80 nm using a focused ion beam (FIB, Auriga, Carl Zeiss, Oberkochen, Germany). Additionally, the tensile tests were conducted using dog-bone specimens with a gauge section of 1 mm (thickness) × 10.16 mm (length) × 3.175 mm (width) at room temperature, oriented perpendicularly to the FB plates, at a loading speed of 10^−3^ m/min (ASME BPVC-IX-2015). The tensile test results for each condition represent the average of three sets of valid data with the same stretching slope to ensure the reliability of the data.

## 3. Results and Discussion

### 3.1. Microstructure Characterization and Analysis

**Figure 1:**
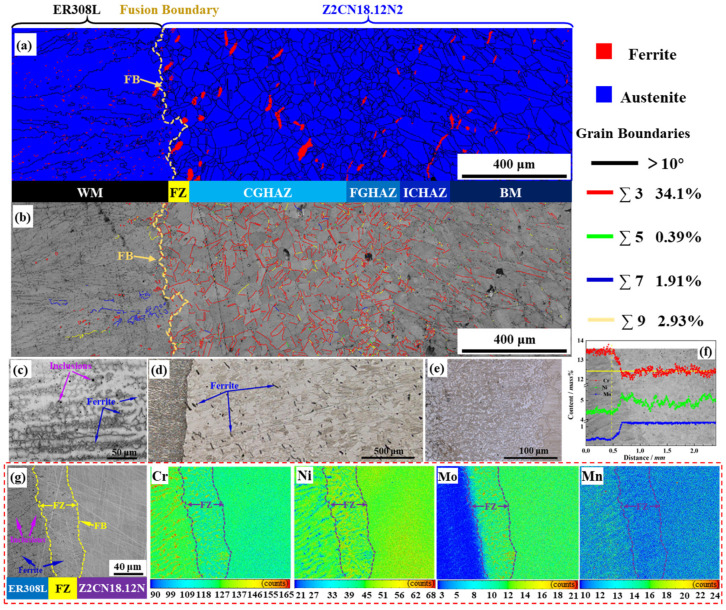
EBSD analyses of phase distribution (**a**) and grain boundary character distribution (**b**) maps of the DMWJs interface; OM of the WM (**c**), DMWJs (**d**), and FZ (**e**) after etching; the composition profile of the DMWJs interface in an unaged condition (**f**,**g**).

**Figure 2:**
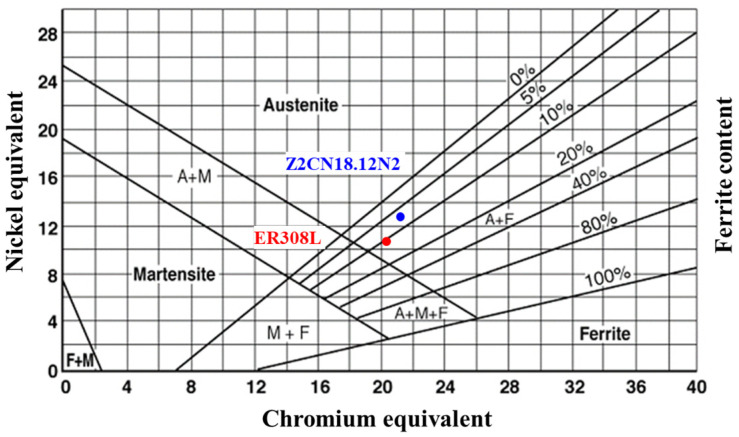
Prediction of microstructure for DMWJs according to the Schaeffler diagram.

Figure 1a demonstrates the cross-sectional image of the welded joint, which consists of weld metal (WM, ER308L), fusion zone (FZ), heat affected zone (HAZ), and base metal (BM, Z2CN18.12N2). The WM microstructure comprises a large number of ferrite phases between the dendritic grain boundaries of austenite. Figure 1c demonstrates vermicular, lathy, needle-like, and globular ferrite morphologies in WM. Additionally, dark dotted inclusions and spherical pores in WM are randomly distributed in grains and along grain boundaries or phase boundaries. The equiaxed austenitic grains are the matrix structure with some annealing twins in the austenite grains. Moreover, a small amount of ferrite is randomly distributed in the austenite grains or along their boundaries, as shown in Figure 1a. The ferrite content of the ER308L and Z2CN18.12N2 can be estimated on the Schaeffler diagram by calculating Cr and Ni equivalent values with the equations Ni_eq_ = 30 C% + 0.5 Mn% + Ni% and Cr_eq_ = Mo% + 0.5 Nb% + 1.5 Si% + Cr% [9,11,25,26]. The Cr_eq_ and Ni_eq_ of ER308L are 20.26 and 11.14, respectively, and the corresponding content is about 10.2 vol% as calculated via the Schaeffler diagram and 8.7~12.1 vol% as calculated using OM. Similarly, the Ni_eq_ and Cr_eq_ of Z2CN18.12N2 are 13.62 and 20.65, respectively, and the corresponding content is roughly 8.3 vol% as calculated via the Schaeffler diagram and 6.3~10.5 vol% as calculated using OM. The Schaeffler diagram is shown in Figure 2.

Figure 1a illustrates that HAZ can be recognized, i.e., coarse-grained HAZ (CGHAZ), fine-grained HAZ (FGHAZ), and inter-critical HAZ (ICHAZ). Furthermore, the HAZ width is approximately 0.8 mm. The microstructure transition from the fusion boundary (FB) to Z2CN18.12N2 is coarse austenite + small amounts of coarse ferrite → a few coarse ferrite + fine austenite → equiaxed austenite + small amounts of fine ferrite. The observed boundaries across the DMWJs can be classified according to the coincidence site lattice (CSL) model. The majority of Σ3 grain boundaries are concentrated within the HAZ or twin boundaries of the Z2CN18.12N2. A few Σ9 grain boundaries are randomly distributed in the Z2CN18.12N2, but the contents of low number CSL boundaries (Σ5, Σ7,) are negligible, as shown in Figure 1b. Sakaguchi et al. [27] proved that Cr depletion (RIS) significantly reduces Σ9 or Σ3 grain boundaries. Additionally, Figure 1f,g present an elemental gradient in the dilution zone near the FB between the ER308L (left) and Z2CN18.12N2 (right), which is the fusion zone.

In the FZ, a network of ferrite distributes between the dendritic grain boundaries of austenite, as shown in Figure 1e,g. As revealed in Figure 1e, the dendritic structures at the interface grew perpendicularly to the FB because of the substantial temperature gradient. It can be noted that there are no significant inclusions or voids in the FZ. According to the elemental distribution of the DMWJs in Figure 1g, the FZ marked with yellow dashed lines is approximately 40 μm. Furthermore, the Cr and Mo distributions correlate with the ferrite distribution, enabling ferrite and austenite differentiation.

**Figure 3:**
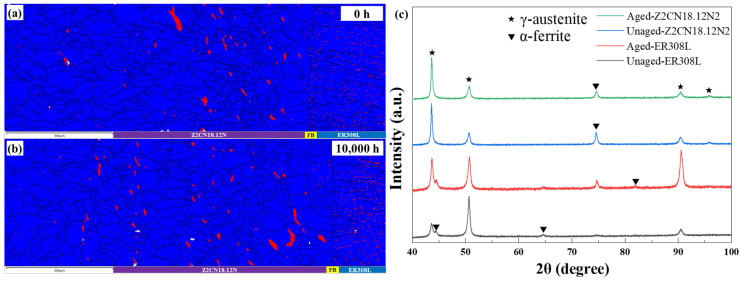
The EBSD analyses of phase distribution across the DMWJs after aging for 0 h (**a**) and 10,000 h (**b**); (**c**) XRD pattern of materials after aging.

After thermal aging, the distribution and size of phases are in accordance with those of the unaged DMWJs specimens in terms of most details. The aged microstructure of Z2CN18.12N2 is composed of equiaxed austenitic grains with some annealing twins in the austenite matrix. Likewise, various morphologies of the ferrite phase are randomly distributed in the austenite grains or along their boundaries. The XRD pattern in Figure 3c shows that Z2CN18.12N2 and ER308L are composed of ferrite and austenite and no other phase precipitates after thermal aging. Figure 3 reveals that the effects of changes in microstructure on the distribution and size of phases are negligible after thermal aging, as shown in the overall macro-views. The ferrite distribution and content remain stable after thermal aging, which follows the previous literature [1].

### 3.2. Mechanical Properties Test Analysis

#### 3.2.1. Nano-Hardness Test and Analysis

**Figure 4:**
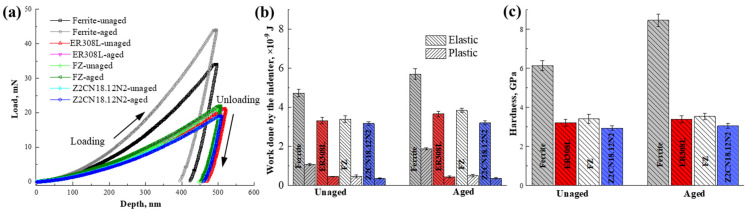
Nanoindentation tests on austenite and ferrite in the DMWJs. (**a**) Load–displacement curves; (**b**) the work carried out by the indenter and (**c**) nanohardness, respectively.

The nanoindentation tests were applied to characterize the micromechanical properties of both the unaged and the 10,000 h aged specimens of DMWJs. The typical load–displacement curves of the DMWJs for the unaged and aged conditions are shown in Figure 4a. The peak of nanoindentation loading in the ferrite phase noticeably grows from 33.97 mN for the unaged station to 43.95 mN after thermal aging. While those in austenite remain nearly at the same level as in DMWJs. Moreover, the residual plastic indentation depths for ferrite reduce from 422.8 nm to 394.5 nm after thermal aging, while those of austenite show no obvious changes. The slope of the ferrite loading curves climbed after thermal aging, and the nano-indentation load of ferrite phases at the same indentation depth grew, demonstrating an increase in strength. In contrast, the slope of the loading curves for austenite in DMWJs remained close to stable after thermal aging.

The work carried out by the indenter is composed of elastic work and plastic work. The elastic work is the area included within the unloading curve and the displacement axis (W_elast_), while the plastic work is the area surrounded by the displacement axis and the loading and unloading curves (W_plast_). Figure 4b shows the changes in elastic and plastic deformation work carried out by the indenter in the unaged and aged DMWJs. For the unaged samples, both the elastic and plastic work performed in ferrite is much higher than the work performed in austenite. Moreover, the elastic and plastic work of austenite display minor differences in different parts of DMWJs, and the following trend can be observed: FZ (3.42 GPa) > ER308L (3.17 GPa) > Z2CN18.12N2 (2.96 GPa). After thermal aging at 400 °C for 10,000 h, the elastic and plastic work carried out in ferrite expands enormously, while that of austenite exhibits little change.

Figure 4c reveals the changes in hardness under the unaged and aged conditions of the DMWJs. The hardness of the ferrite phase dramatically increases from 6.13 GPa in the unaged condition to 8.46 GPa in the 10,000 h aged condition. In contrast, the hardness increases in austenite are negligible. The nanoindentation results demonstrate that the thermal aging effect on the mechanical properties are confined to the ferrite phase, which is in accordance with the previous pieces of literature [28,29,30].

In general, indentation results are mainly subject to the local microstructure; the shape and distribution of ferrite in austenite affect the hardness results [31,32]. At the time, the inclusions and voids dispersed in the phase grains and along grain boundaries in the ER308L decrease the hardness contribution of ferrite to austenite. As for Z2CN18.12N2, when ferrite is dispersed throughout the austenite grains and along their boundaries, the contribution to the hardness of austenite is negligible.

On the other hand, the microstructure evolutions, spinodal decomposition, and G-phase precipitation lead to the embrittlement of the ferrite phase after long thermal aging [8,33,34,35]. Although the literature has proved that microhardness has a linear relation with the variations in ferrite decomposition after thermal aging in ER308L [21,36] and Z2CN18.12N2 [37,38], few reports are concerned with the effect of thermal aging on the microstructure of FZ in DMWJs. The complex ferrite and few inclusions or voids in the FZ suppress the deformation of austenite during the indenter pressing, which promotes the increase in hardness in austenite. Hence, microstructure evolution in aged FZ was observed by TEM and APT, as shown in Figure 4.

**Figure 5:**
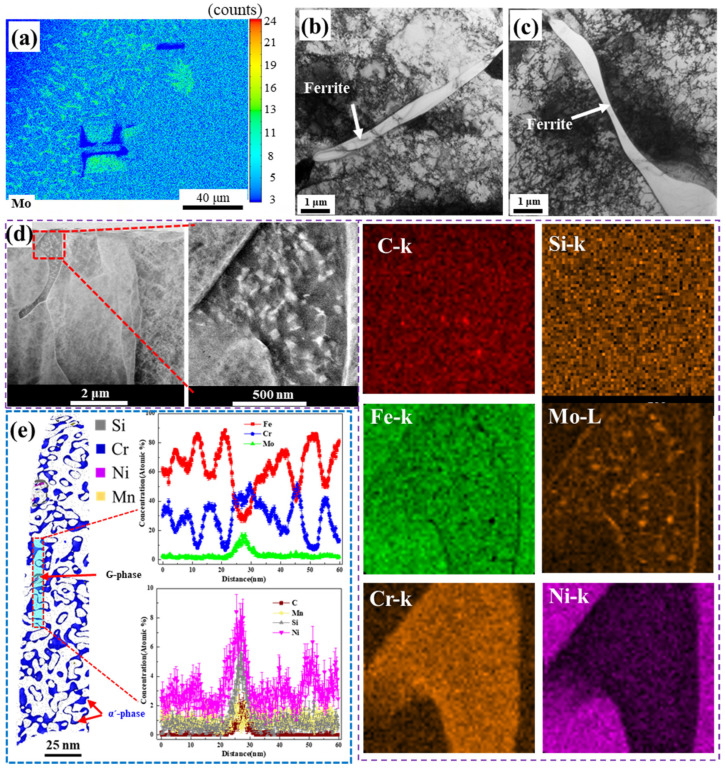
The location of TEM samples prepared by FIB (**a**); typical TEM images in FZ after aging for 0 h (**b**) and 10,000 h (**c**); TEM images and the corresponding element maps of C, Si, Fe, Mo, Cr, and Ni of the FZ after aging for 10,000 h (**d**); 3D APT reconstruction showing composition analysis of the ferrite phase in the FZ of DMWJs after 10,000 h of aging (**e**).

Because the Cr and Mo distributions correlate with the ferrite distribution, enabling ferrite and austenite differentiation, the location of TEM and APT samples can be selected based on the distribution of Cr. The elastic and plastic work of austenite in FZ is higher than BM and WM, as the high cooling rate in the FZ during welding leads to a high dislocation density in austenite, in addition to residual stress (as shown in Figure 5b,c) [29]. On the other hand, the ferritic phase in the FZ undergoes spinodal decomposition (ferrite turns into Cr-rich α’phase and Fe-rich α phase) and G-phase precipitation (Ni_16_Si_3.5_ (Fe, Cr) _3.5_Mn_6_) during the thermal aging process (as shown in Figure 5e) [39,40]. Meanwhile, Ni promotes the spinodal decomposition kinetics in spite of the function of Mo or Mn [5,41]. Lach T.G. et al. [41] reported that rising Mo content enormously accelerated the extent of spinodal decomposition. Therefore, adding Mo and Mn hastens the precipitation of G-phase [3]. Furthermore, thermal segregation enriches Mo at grain boundaries (GBs) [4,42,43,44], as shown in Figure 5d.

**Figure 6:**
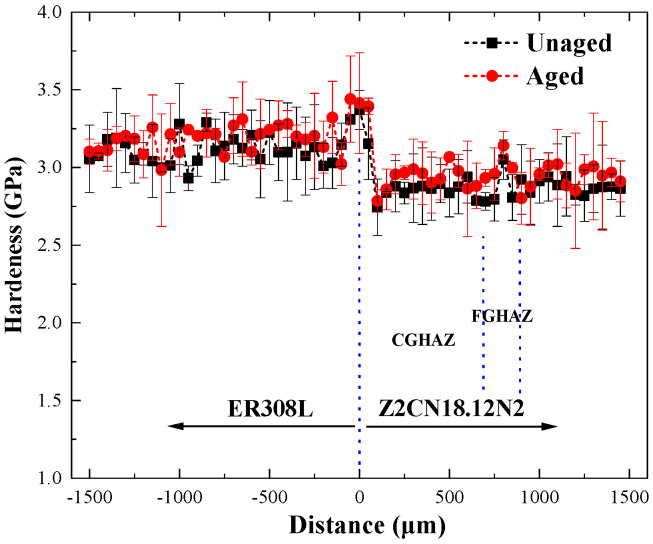
Microhardness of the DMWJs.

The curves of microhardness in the DMWJs under different thermal aging conditions are shown in Figure 6. The average microhardness of DMWJs illustrates that there are obvious differences in mechanical properties between the BM and the WM. Hardness presents the following trend: FZ > ER308L > FGHAZ > BM > CGHAZ. The result is consistent with Figure 3. A softer zone was found in Z2CN18.12N2 near the FB that may result from tempering during welding progress.

As for FGHAZ, grain refining improves the hardness of the BM because higher grain boundary intensity will inhibit dislocation motion by providing more obstacles [11]. Hall E.O. has proved that the hardness is in inverse proportion to the grain diameter [45]. In addition, the coarse grain size near the FB promotes the stability of the austenite. The hardness of DMWJs increases a little after thermal aging, as shown in Figure 6.

#### 3.2.2. Tensile Behavior of the DMWJs

**Figure 7:**
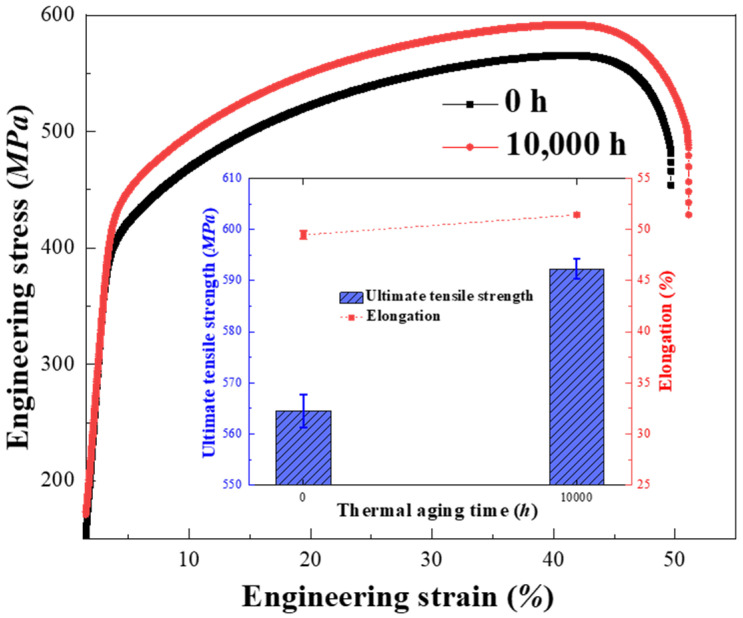
The engineering stress–strain curves of the DMWJs.

Figure 7 illustrates the engineering stress–strain curves of the DMWJs with different thermal aging times. There is no clear yield platform in the curves, but the yield strength increases a little after aging. Additionally, the ultimate tensile strength significantly grows from 564.5 MPa in the unaged condition to 592.7 MPa after ageing for 10,000 h. Nevertheless, there was a minor improvement in the total elongation, and the elongation increased from 49.6% to 51.1% after thermal aging.

**Figure 8:**
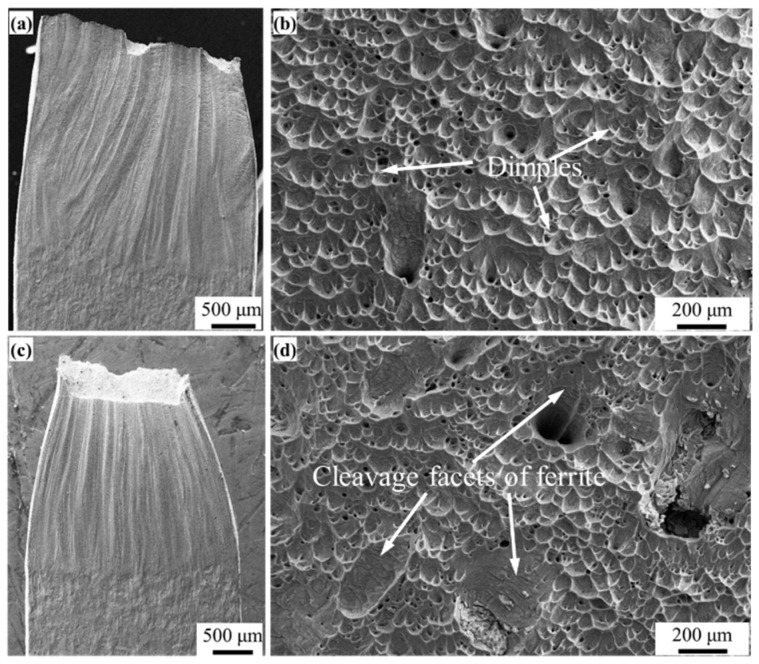
Tensile fracture surface of unaged (**a**) and aged (**c**) specimens of the DMWJs, and fracture morphologies of unaged (**b**) and aged (**d**) specimens.

As illustrated in Figure 8a,c, the fracture location occurred on the ER308L side, attributed to inclusions, voids, and complex ferrite phases (as shown in Figure 1c) formed during welding that enable preferential cracking in ER308L [21,22]. BM exhibits superior fracture toughness compared to WM, regardless of thermal aging time. Austenite acts as though it has been torn apart and plays a role in holding elongation as a soft phase during the fracture process [46]. The ferrite, in the hardening phase, can play a role in maintaining the austenite matrix and improving the tensile strength of materials. At the same time, the increase in ferrite strength promotes the tensile strength of all the DMWJs.

Both ferrite and austenite have good deformation capacity for unaged specimens, as shown in Figure 8a,b. The fracture morphology of the unaged condition does not tear off, many fine dimples are found in the specimen, illustrating ductile fracture features, as shown in Figure 8b. After thermal aging, the sizes of fine dimples are almost unchanged, but the tear edge characteristics increased significantly. According to the results of Figure 4 and Figure 5, there is no obvious change in the strength and plasticity of austenite, but the plasticity and toughness of ferrite decrease after thermal aging. In other words, the plastic deformation of ferrite decreases, and the characteristics of quasi-cleavage are observed in the specimen, as shown in Figure 8d. The above results show that the fracture mode of DMWJs changes from ductile fracture into the mixed fracture mode of ductile fracture and quasi-cleavage fracture after thermal aging. Because ER308L has many defects, such as inclusions and voids, these defects become the sources of cracks in the process of tension, causing the material to fracture preferentially on the ER308L side of DMWJs. Hence, the surface defects and element distribution of ER308L before and after tensile deformation are characterized by EPMA, as shown in Figure 7.

**Figure 9:**
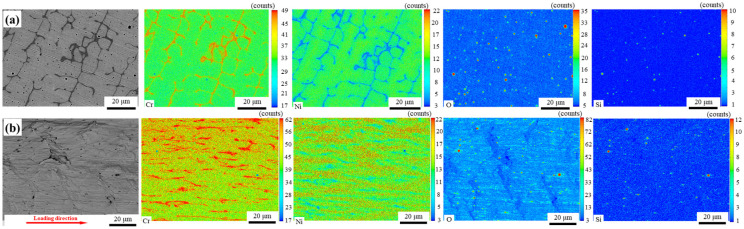
EPMA mapping of the ER308L surface before (**a**) and after (**b**) tensile deformation under unaged conditions.

**Figure 10:**
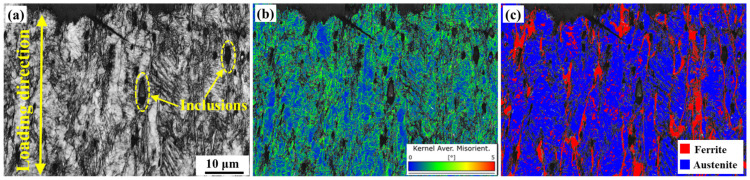
The SEM (**a**) and corresponding EBSD results (step size = 50 nm) of the crack tip on the ER308L: KAM map (**b**); phase distribution (**c**).

Figure 9a shows that the inclusions circled yellow dotted line are Si-rich oxides formed during the welding process. The mechanism of plastic deformation of materials is dislocation movement. Local strain distribution in the crack tip was assessed via kernel average misorientation (KAM), and the KAM map illustrates high dislocation density near the inclusions and ferrite, as shown in Figure 10. When dislocations meet inclusions (hard phase) during the deformation process, stress concentration easily occur near them, forming crack sources. As the deformation increases, cracks form near the inclusions, as shown in Figure 9b.

## 4. Conclusions

In the present work, the dissimilar ER308L/Z2CND18.12N2 welds were investigated in order to understand the microstructure evolution and mechanical properties after thermal aging at 400 °C for 10,000 h. The mechanical properties were evaluated using a nano-indenter. Microstructure evolution in the fusion zone was examined using TEM and APT. The following conclusions can be drawn:

1. The fusion zone with the chemical composition gradient was about 40 μm from the Z2CND18.12N2 to ER308L.
2. The CSL boundaries were mainly concentrated in the HAZ of Z2CND18.12N2.
3. Significant spinodal decomposition and G-phase precipitation were observed for the first time in the thermally aged ferrite phase of the fusion zone.
4. The hardness distribution along the DMWJs was non-uniform, and the average microhardness presents the following trend: FZ > ER308L > FGHAZ > BM > CGHAZ.
5. The ultimate tensile strength of the DMWJs significantly increases from 564.5 MPa in the unaged condition to 592.7 MPa to MPa after thermal aging.
6. Irrespective of thermal aging time, Z2CND18.12N2 exhibited superior fracture toughness in comparison to ER308L.
7. Fractures occurred preferentially in the ER308L, and the fracture mode changed from ductile fractures to ductile + quasi-cleavage fractures.

## Figures and Tables

**Table 1:** Chemical compositions (wt.%) of the ER308L and Z2CND18.12N2.

Materials	C	Cr	Ni	Mo	Mn	Co	P	S	Cu	Si	Fe
ER308L	0.027	19.22	10.62	0.19	2.02	0.13	0.009	0.001	-	0.013	Bal.
Z2CND18.12N2	0.022	17.74	11.99	2.26	1.93	0.09	0.022	0.009	0.09	0.431	Bal.

## Data Availability

The data presented in this study are available on request from the corresponding author.
